# Comprehensive genomic profiling of small cell lung cancer in Chinese patients and the implications for therapeutic potential

**DOI:** 10.1002/cam4.2199

**Published:** 2019-06-14

**Authors:** Jing Hu, Yu Wang, Yuan Zhang, Yanfei Yu, Hui Chen, Kuai Liu, Ming Yao, Kai Wang, Weiguang Gu, Tao Shou

**Affiliations:** ^1^ Department of Medical Oncology First People's Hospital of Yunnan Province Kunming China; ^2^ Department of Medical Oncology The Affiliated Hospital of Kunming University of Science and Technology Kunming China; ^3^ Tumor Research and Therapy Center Shandong Provincial Hospital Affiliated to Shandong University Ji'nan China; ^4^ OrigiMed Shanghai China; ^5^ Precision Medicine Center Shulan (Hangzhou) Hospital Hangzhou China; ^6^ Department of Medical Oncology Southern Medical University Nanfang Hospital Foshan China; ^7^ Department of Medical Oncology People's Hospital of Nan Hai District Foshan China

**Keywords:** comprehensive genomic profiling, next generation sequencing, small cell lung cancer, targeted therapy, tumor mutation burden

## Abstract

**Background:**

Small cell lung cancer (SCLC) is one of the deadliest malignancies and accounts for nearly 15% of lung cancers. Previous study had revealed the genomic characterization of SCLC in Western patients. However, little is known about that in Chinese SCLC patients.

**Methods:**

Formalin‐fixed paraffin‐embedded tumor tissues and matched blood samples from 122 Chinese SCLC patients were collected for next generation sequencing to detect 450 cancer‐related genes. All pathological diagnoses were confirmed by independent pathologists.

**Results:**

The most frequently altered genes were *TP53* (93.4%), *RB1* (78.7%), *LRP1B* (18.9%), *KMT2D* (15.6%), *FAT1* (11.5%), *KMT2C* (11.5%), *SPTA1* (11.5%), *STK24* (11.5%), *FAM135B* (10.7%), and *NOTCH1* (10.7%). The gene fusion/rearrangement detection rate was 16.4%, and mostly occurred in chromosomes 7 and 17. The rate of co‐occurring mutations of *TP53* and *RB1* in these Chinese SCLC patients was 74.6%, and lower than the reported Western patients (90.9%, *P* = 0.007). The most common gene mutations (83.6%) were found in cell cycle signaling pathway in Chinese SCLC patients. Mutation of Wnt and Notch signaling pathways in the Chinese cohort were lower than Western cohort (*P* = 0.0013 and 0.0068). A significant association was found between high tumor mutation burden and mutations involved in *FAT1*,* TP53*,* SPTA1*,* KEAP1*,* KMT2D*,* MAGI2*,* NOTCH2*,* NOTCH3*,* FLT1*,* KDM6A*, and *FAT4*.

**Conclusions:**

In this study, we characterized the genomic alterations profile of Chinese SCLC patients. Compared with westerners, the genetic alterations of Chinese SCLC patients presented different patterns. Our data might provide useful information in targeted therapy and drug development for Chinese SCLC patients.

## 1 INTRODUCTION

Lung cancer is the leading cause of cancer‐related deaths worldwide. Small cell lung cancer (SCLC) accounts for nearly 13% of lung cancer.^1^ SCLC patients can be divided into limited disease (LD) and extensive disease (ED). Patients with LD receive chemotherapy or/and radiation therapy, and their median survival is 16‐24 months.^2^, ^3^ ED accounts for the majority of SCLC. Chemotherapy remains the cornerstone of treatment, while radical thoracic radiotherapy is not suitable treatment option for ED patients. ED has a high recurrence rate, and the median survival is usually 7‐12 months.^2^, ^3^

Many clinical trials have demonstrated that the overall response rate and progression‐free survival are improved by using targeted therapy compared to the traditional chemotherapy.^4^, ^5^, ^6^ In the past few decades, there are 11 types of oncogenic protein kinase inhibitors, including Bcr‐Abl tyrosine kinase inhibitors, epidermal growth factor receptor tyrosine kinase inhibitors, and vascular endothelial growth factor receptor tyrosine kinase inhibitors, etc which have been approved by FDA for cancer patients treatments.^7^ Thus, identifying the key onco‐driven gene alterations become the critical determinant for clinicians to choose potential targeted therapy. Data from The Cancer Genome Atlas revealed that SCLCs are highly genomically complex,^8^ and this genomic complexity of malignant cells caused by contentious gene alterations requires us to constantly explore new molecular alterations and potential therapeutic targets for patients. Several driven gene alterations of lung cancer have been identified for active targeted therapeutics. Alterations in *TP53*, *RB1*, and *MLL2* are the most in SCLC.^9^ Others include *RICTOR* (10%), *KIT* (7%), *PIK3CA* (6%), *EGFR* (5%), *PTEN* (5%), *KRAS* (5%), *MCL1* (4%), *FGFR1* (4%), *BRCA2*, (4%), *TSC1* (3%), *NF1* (3%), *EPHA3* (3%), and *CCND1* were identified in SCLC as well.^9^ Amplifications of *MYC* family members can be found in about 22% of SCLC patients and 50% of SCLC cell lines.^10^ In addition, bi‐allelic inactivation of *TP53* and RB1 can be detected in nearly all the SCLC tumors, suggesting that loss of the tumor suppressors *TP53* and *RB1* is obligatory in SCLC.^11^ The development of next generation sequencing (NGS) technology and platforms permit rapid and precise identification of oncogenic alterations.^12^ Comprehensive genomic profiling of tumors based on NGS technology can detect all kinds of genetic changes from point mutations to large structural variations in lung cancer. In this study, we ran NGS on 122 Chinese SCLC patients tumor sample and analyzed the gene sequencing data. We identified some unique genetic variant features in Chinese SCLC patients and further compared these features to the Western SCLC patients reported previously. In addition, we found that somatic co‐occurring *TP53*/*RB1* mutations were frequent in Chinese SCLC patients. We further identified gene alterations involved in several signaling pathways and several targetable gene variations including homologous recombination (HR), FGF family, *KIT*,* PDGFRA*, and *DDR2*. These results might be helpful in the selection of potential targeted therapeutic options and predicting prognosis.

## 2 MATERIALS AND METHODS

### 2.1 Subjects inclusion and samples collection

This study was approved by the Institution Review Board according to the Declaration of Helsinki and obtained the informed consent from all enrolled patients. A total of 122 Chinese patients who received surgery or biopsy with a final pathological diagnosis of SCLC from December 2016 to June 2018 were enrolled in this study. All patients were diagnosed with pathological, imaging, and clinical findings evidence. Both formalin‐fixed paraffin‐embedded (FFPE) tumor tissues and matched blood samples from these 122 Chinese SCLC patients were collected and transferred to the OrigiMed, Shanghai for genetic alteration detection. Genomic DNA of tumor samples and white blood cells from match blood was extracted from the FFPE and blood samples using QIAamp DNA FFPE Tissue Kit and QIAamp DNA Blood Midi Kit (Qiagen, Hilden, Germany) according to the manufacturer's instructions. The concentration of DNA was measured by Qubit and normalized to 20‐50 ng/μL.

### 2.2 Detection of genomic alterations

The genomic alterations were examined and the profile was produced using the YuanSu^TM^450 gene panel (OrigiMed, Shanghai, China). This panel covers all the coding exons of 450 cancer‐related genes and 64 selected introns of 39 genes that frequently rearranged in solid tumors. The genes were captured and sequenced with a mean coverage of 800× using an Illumina instrument, which can detect and analyze the point mutation, insertion/deletion, gene copy number variation and gene rearrangement/fusion (including large, >100 bp fragment insertion/deletion), and other variant forms related to cancers at DNA level. White blood cells isolated from whole blood were used as matched normal control. For germline mutations, common SNPs defined as those from dbSNP database (version 147), or frequency over 1.5% of Exome Sequencing Project 6500 (ESP6500) or over 1.5% of 1000 genome project were excluded.

### 2.3 Statistical analysis

The qualitative variables were analyzed by Fisher's exact test. The comparisons of normal quantitative distributed data were performed using *t* test, and Wilcoxon rank test for the non‐normal distributed data. All of the statistical analyses were performed with SPSS 22.0.

## 3 RESULTS

### 3.1 Patients' characteristics

Nighty‐seven (97) male and 25 females SCLC patients were included in this study. Age of male SCLC patients was in the range of 29 to 83 with median age 61 years old. The female patients were from 47 to 76 with median age 63 years old. The detailed patients' characteristics including sex, ages and other major testing data are summarized in Table 1.

**Table 1:** The characteristics of the participants

Characteristics	N	Percentage
Age		
＜65 y	46	37.7
≥65 y	76	62.3
Sex		
Male	97	79.5
Female	25	20.5
Smoking status		
Present or former smoker	44	36.1
Non‐smoker	36	29.5
Unknown	42	34.4
Subtype		
SCLC	111	91.0
Combined SCLC	11	9.0
Source of samples		
Primary	107	87.7
Metastases	15	12.3
MSI status		
MSS/MSI‐L	111	91.0
MSI‐H	0	0.0
Unsure	11	9.0
TMB status		
<10 muts/Mb	66	54.1
≥10 muts/Mb	56	45.9
10‐16 muts/Mb	37	30.3
16‐20 muts/Mb	8	6.6
≥20 muts/Mb	11	9.0
Stages		
Ⅰ	8	6.6
Ⅱ	4	3.3
Ⅲ	14	11.5
Ⅳ	25	20.5
Unknown	71	58.2
Total	122	

Abbreviations: SCLC, small cell lung cancer; TMB, tumor mutation burden.

### 3.2 Comprehensive genomic profiles of SCLC

**Figure 1:**
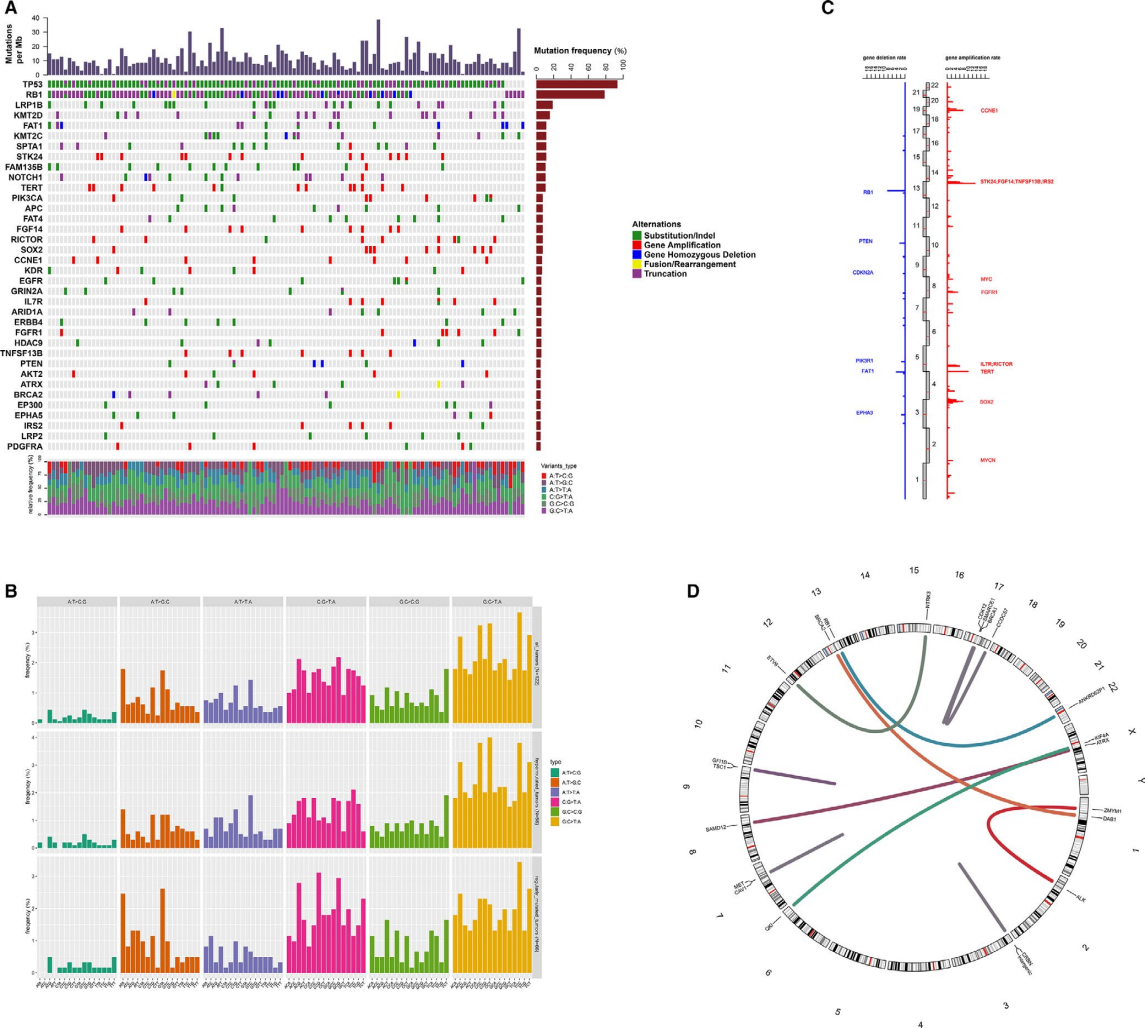
Summary of gene mutations in 122 Chinese small cell lung cancer (SCLC) patients. A, Genomic profiling. The abscissa was the tumor specimens and the ordinate was the gene names. Genetic alterations annotated according to the color panel on the right side of the image. B, Single nucleotide variation analysis. C, The frequencies of copy number variations of all chromosomal changes. The numbers of 1‐22 in the middle represented the human chromosome number. The names of the 12 genes with the highest amplification magnitude (red) and six genes with the highest deletion magnitude (blue) were marked. D, Gene fusion/arrangement analysis

All data from these 122 patients' samples were analyzed. Among them, 119 were found to have clinical relevant genomic alterations (CRGAs), two were negative, and one was found to have unknown clinical significance variants. Ranked Top 10 genes with most mutation frequency were *TP53* (N = 114, 93.4%), *RB1* (N = 96, 78.7%), *LRP1B* (N = 23, 18.9%), *KMT2D* (N = 19, 15.6%), *FAT1* (N = 14, 11.5%), *KMT2C* (N = 14, 11.5%), *SPTA1* (N = 14, 11.5%), *STK24* (N = 14, 11.5%), *FAM135B* (N = 13, 10.7%), and *NOTCH1* (N = 13, 10.7%) (Figure 1A). The single nucleotide variation (SNV) was dominated by C>A, which accounting for 36.2% of the total SNVs. Among them, the two highest frequencies were TCC>TAC and TCT>TAT. Others, C>G was 12.9%, C>T was 24.4%, T>A was 10.9%, T>C was 12.4%, and the lowest frequency of SNV T>G was 3.2%. The SNV profiles were slightly different in the population with high‐tumor mutation burden (TMB‐H, TMB ≥10) and low TMB (TMB‐L, TMB <10). The frequency of C>A in Chinese SCLC patients with TMB‐H was higher than that in TMB‐L (39.5% vs. 31.0%，*P* = 0.0006); while the frequency of C>T in the patients with TMB‐H was lower than that in TMB‐L (21.7% vs. 28.9%, *P* = 0.0010) (Figure 1B). Copy number variation (CNV) analysis showed that the most frequently amplified genes were *STK24* (13.1%), *TERT* (9.8%), *CCNE1* (7.4%), *FGF14* (7.4%), and *SOX2* (7.4%); the most frequently deleted genes were *RB1* (8.2%), *FAT1* (4.1%), *PTEN* (2.46%), *EPHA3* (1.6%), and *PIK3R1* (1.6%) (Figure 1C). Among these 122 cases of Chinese SCLC patients, gene fusion/rearrangement was detected in 20 cases. Multiple gene rearrangements were detected in four SCLC patients. The fusion/ rearrangement mostly occurred in chromosomes 7 and 17, and 43 genes including *ALK*,* BRCA1*,* MET*, and *NTRK3* involved in the fusion/rearrangement. The clinical relevant gene arrangements, such as *ETV6‐NTRK3* and *CCDC57‐BRCA1*, were found in 12 patients, among which *ETV6*‐*NTRK3* fusion was detected in 1.6% (N = 2) of SCLC patients (Figure 1D). Interestingly, both of patients with *ETV6*‐*NTRK3* fusion are TMB‐H, one case is 17.6 Muts/Mb and the other is 48.8 Muts/Mb. For the patients with TMB = 48.8 Muts/Mb, ETV6‐NTRK3 fusion is the only driver mutation, while for another patients with TMB = 17.6 Muts/Mb, we also detected EGFR exon 19‐del and EGFR amplification. All mutations in these two patients are listed in Table S1.

For germline mutations, we identified six truncation variants in five patients, including two cases with BRCA1 (S1040Afs*8) and BRCA2 (T1087Qfs*5) variants, one case with RAD51C (E45fs*) variant, one with TP53 (G266*) variant, and one with TP53 (A88Gfs*32) and RB1 (W681*) truncation variants.

### 3.3 Co‐occurring mutations of TP53 and RB1

**Figure 2:**
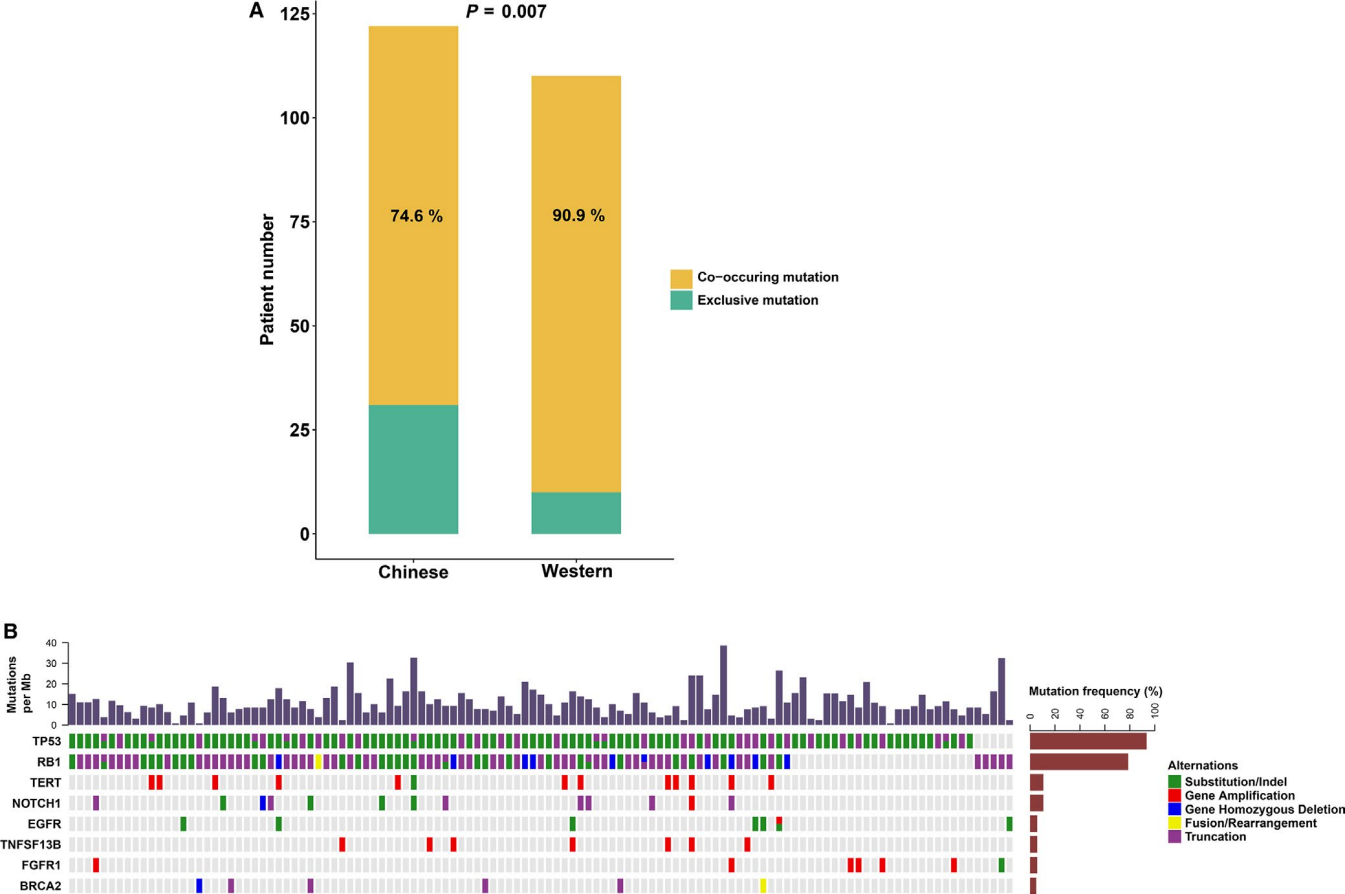
Analysis of *TP53/RB1* co‐alterations in 122 Chinese patients with small cell lung cancer (SCLC). A, Comparison of detection rate between Chinese and Western SCLC patients. B, Genes which had different mutation characteristics in Chinese patients with SCLC carrying both *TP53* and *RB1* mutations and *TP53* mutation only

Previous reports showed that most of the SCLC tumors contain co‐occurring *RB1* and *TP53* missense mutations.^11^, ^13^ Here, we further analyzed the co‐occurring mutations of *TP53* and RB1 in these Chinese SCLC patients. The results revealed that 74.6% of Chinese SCLC patients harbored both *TP53* and *RB1* mutation, which was lower than that reported by an international group from Europe and US which we speculated that the patients were mainly the Western population (90.9%, *P* = 0.007) (Figure 2A).^11^ The data from previous study were calculated based on oncoprint results in the literature: of the 110 samples, 100 had both *TP53* and *RB1* mutations. Mutation of *EGFR* (7.4%), *NOTCH1* (11.8%), *TNFSF13B* (6.4%) and *BRCA2* (5.5%), as well as *TERT* amplification (10.9%) were restricted in *TP53*/RB1 co‐mutation samples, while *FGFR1* amplification was more common in the SCLC patients with *TP53* mutation alone (4/12 vs 2/93, *P* < 0.001) (Figure 2B).

### 3.4 Detection of gene mutations in cancer‐related signaling pathways

**Figure 3:**
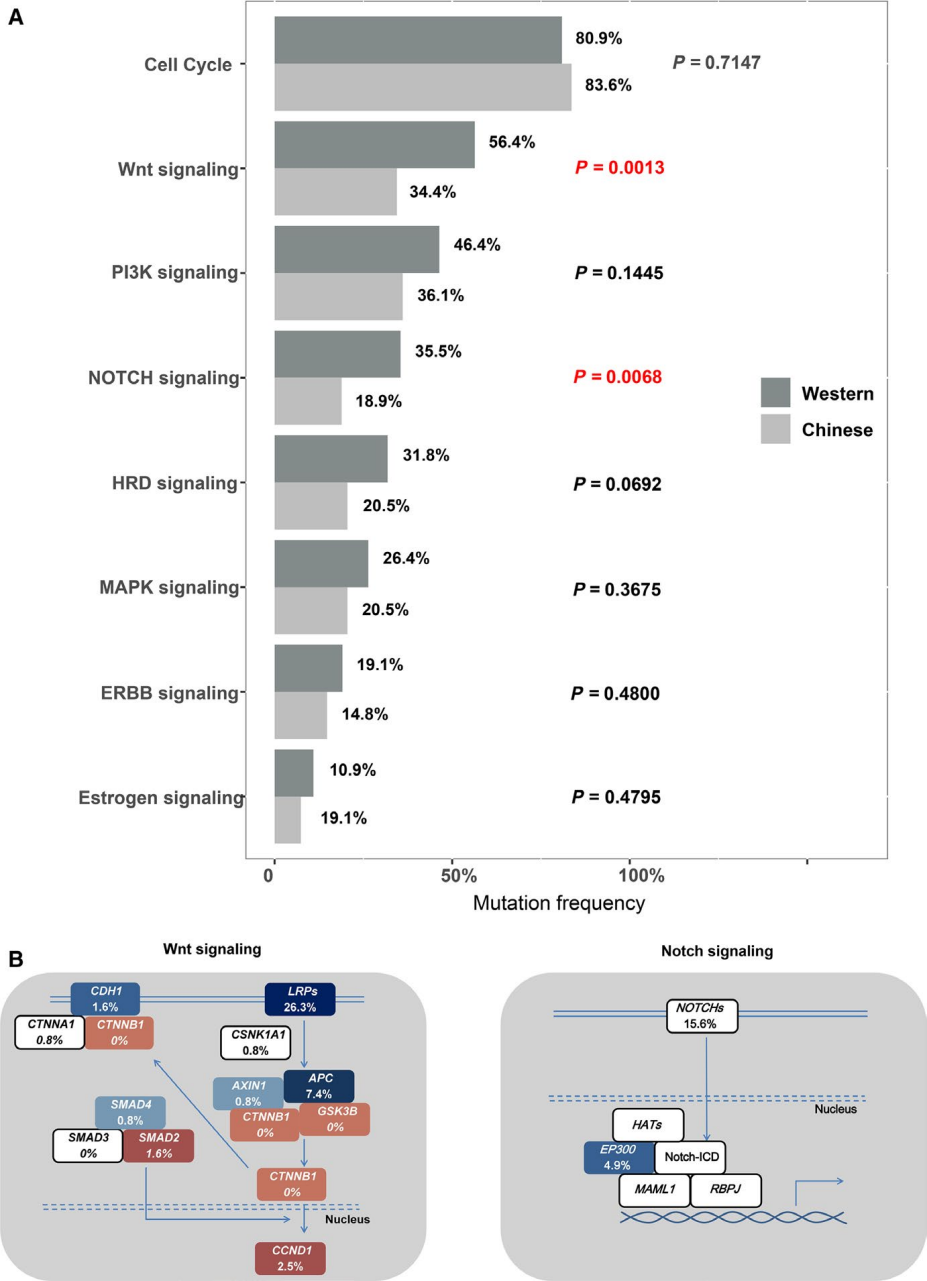
Analysis of mutations involved in signaling pathways in 122 Chinese patients with small cell lung cancer (SCLC). A, Comparison of detection rate of gene mutations in the pathways between Chinese and Western patient. B, Alterations in Wnt and Notch signaling pathways genes. The brown and blue boxes represent genes with activation and inactivation alterations, respectively. The number in each box represents the frequency of each genes in Chinese cohort

The gene mutations of some SCLC‐related pathways were analyzed. The related genes in these signaling pathways included in YuanSu^TM^450 panel are listed in Table S2. Gene mutations in cell cycle, Wnt and PI3K signaling pathways were the most common in these Chinese SCLC patients. Among these mutations, the number of cell cycle signaling pathway‐related gene mutations was the largest, accounting for 83.6% of all the SCLC patients. Compared with the previous study,^11^ the frequencies of gene variations in Wnt and Notch signaling pathways in Chinese patients were significantly lower than that in Western patients, respectively (*P* = 0.0013; 0.0068) (Figure 3).

### 3.5 Tumor mutation burden analysis

**Figure 4:**
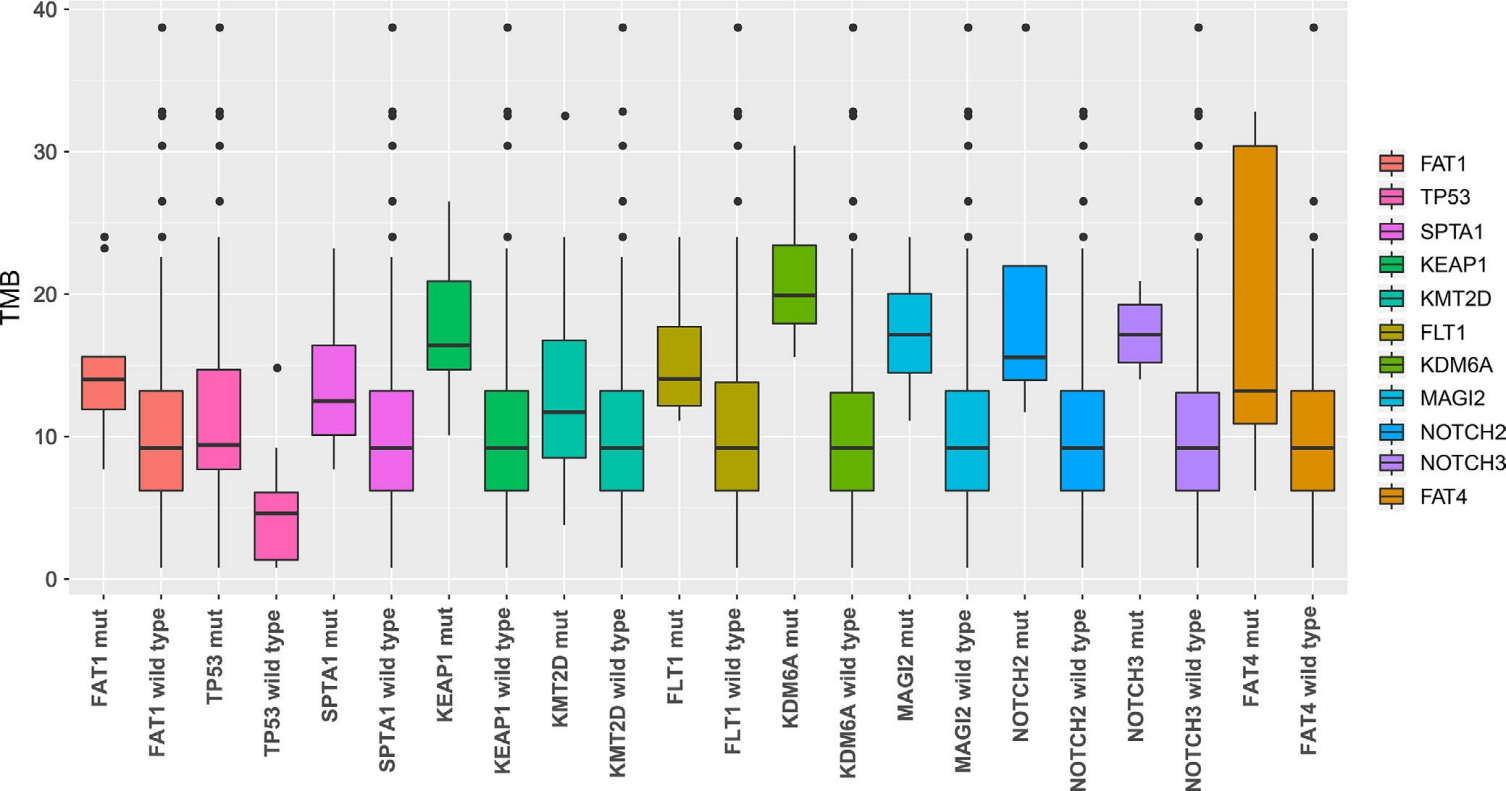
Comparison of median TMB in Chinese SCLC patients with certain specific gene mutations. SCLC, small cell lung cancer; TMB, tumor mutation burden

By Fisher's exact test, we found a significant association between TMB‐H and multiple gene mutations, including *FAT1*,* TP53*,* SPTA1*,* KEAP1*,* KMT2D*,* MAGI2*,* NOTCH2*,* NOTCH3*,* FLT1*,* KDM6A*, and *FAT4*, in the Chinese SCLC patients (Table S3). The mutation rates of the genes mentioned above in TMB‐H group were much higher than that in TMB‐L group. In addition, the median TMB of patients with these mutations was higher than those without the mutations (Figure 4).

### 3.6 Correlation between gene mutation and clinical stages of disease

We compared the differences of mutation rates of the related genes between the stages III/IV and stages I/II of SCLC patients. To determine whether the mutation of a gene is related to the clinical stage of SCLC, the statistical analysis was performed using Fisher's exact test and further correction was performed using the Benjamini‐Hochberg method. *EPHA3* and *NOTCH2* gene mutations are more prevalent in stage I/II SCLC patients, while TP53 mutations are more common in stage III/IV SCLC patients. After Benjamini‐Hochberg correction, no statistical differences were observed in any genes between the stages III/IV and stages I/II of SCLC patients (Table S4), suggesting that gene mutations occur early in tumorigenesis and are somewhat conserved during tumor growth.

### 3.7 Analysis of Notch, CREBBP, and EP300 gene alterations

**Figure 5:**
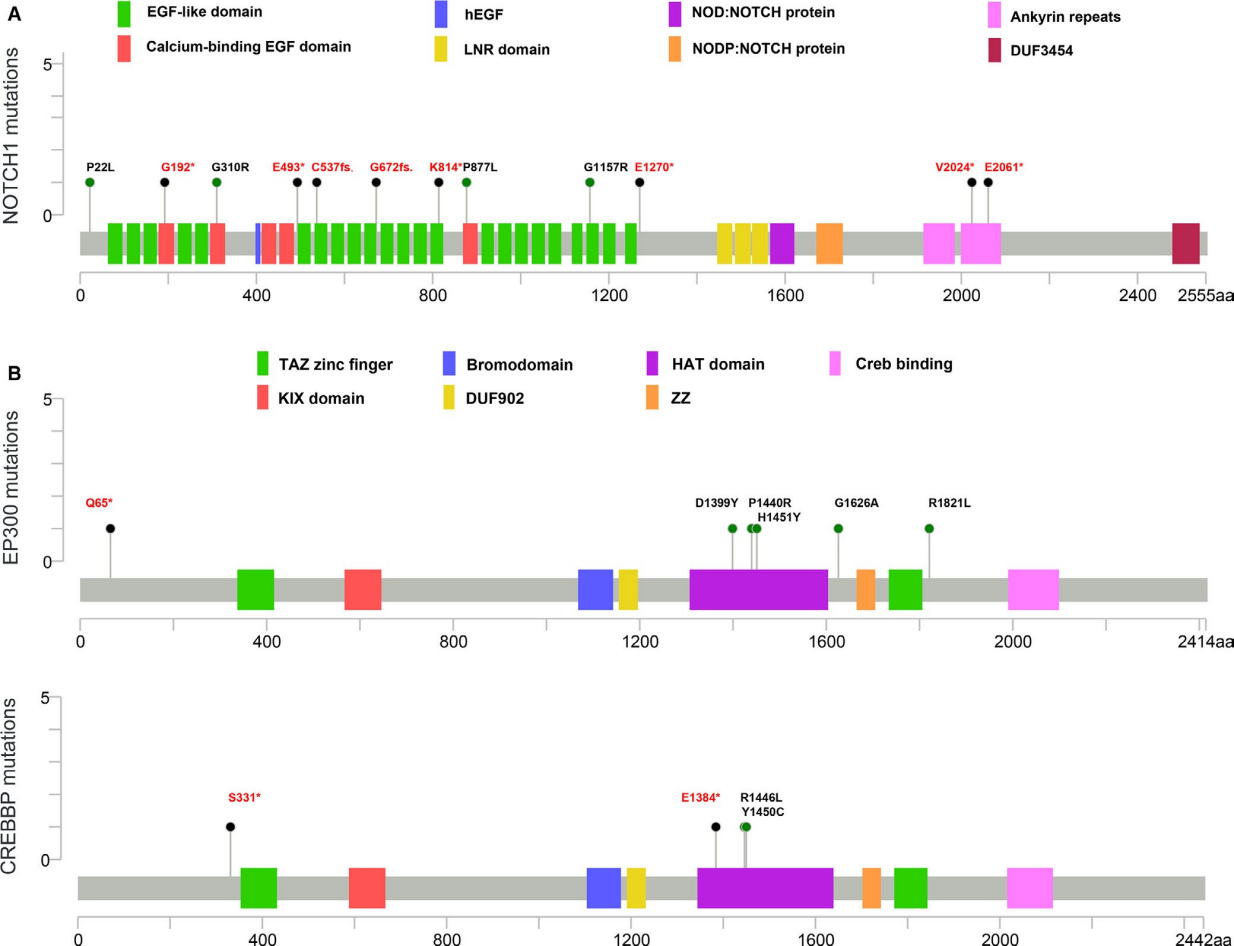
The distribution of mutations of *Notch1*, *EP300*, and *CREBBP* identified in Chinese small cell lung cancer (SCLC) patients. A, *Notch1* mutations identified in Chinese SCLC patients mainly occurred in the EGF‐like domain or calcium‐binding EGF domain. B, Clinical relevant genomic alterations of *EP300* and *CREBBP* mostly clustered in the HAT domain

Of the 122 SCLC patients, 35 had *NOTCH* family gene mutations and seven patients had at least two forms of mutations. Of all the genes variations, the CRGAs accounted for 15.6% (19/122). *Notch1* mutation was 10.7% (13/122), both *Notch2* and *Notch3* mutations were 3.3% (4/122), and *Notch4* mutation was 0.8% (1/122). Twelve SNV mutations, including four substitution mutations and eight truncated mutations, were detected in 13 patients with the *Notch1* mutations (Figure 5A). About 66.7% (8/12) of the SNVs occurred in the EGF‐like domain or Calcium‐binding EGF domain. EP300 mutation was detected in nine patients, of which 4.9% (6/122) was the CRGAs. The *CREBBP* gene mutation was detected in five patients, and they were all the CRGAs. CRGAs occurring in the *CREBBP* and *EP300* genes mostly clustered in the HAT domain (Figure 5B), accounting for 60% of all SNVs. The mutation frequencies of *Notch*,* CREBBP*, and *EP300* were much lower than those of the reported previously.^11^

## 4 DISCUSSION

The widely used comprehensive hybrid capture‐based NGS technology makes it possible to detect the driven gene mutations comprehensively in cancer patients' samples. Targeted therapy for patients who harbor specific drive gene alterations can prolong their survival compared with patients without targeted therapy. Therefore, continuous in‐depth studies of oncogene mutation have significant value for understanding both the biological characteristics of cancer cells and clinical treatment optimization. SCLC is a highly heterogeneous rapidly lethal disease with few therapeutic options. The primary objective of our study is to understand the genomic profile of SCLC in Chinese patients and compare it with Western patients, and further intend to provide new information for therapeutic approaches that will be suitable to Chinese SCLC patients.

In this study, we found a large number of genes with point mutations, structural variants, and fusions. The genes with the highest mutation frequency were *TP53*, *RB1*, *LRP1B*, *KMT2D*, *FAT1*, *KMT2C*, *SPTA1*, *STK24*, *FAM135B*, and *NOTCH1*. For CNV, the most frequently amplified genes were STK24, TERT, CCNE1, FGF14, and SOX2, and the most frequently deleted genes were RB1, FAT1, PTEN, EPHA3, and PIK3R1. Most of these gene mutations correlated with poor prognosis in cancer, but have no targetable drugs, may explain the poor survival of SCLC.^14^, ^15^, ^16^, ^17^, ^18^, ^19^ However, we defined several subgroup patients who may benefit from genotyping and subsequent targeted therapy. ETV6‐NTRK3 fusion was detected in 1.6% (2/122) of SCLC, which was relatively rare, but may suggest a response to pan‐NTRK as well as ALK and ROS1 tyrosine kinase inhibitors. In addition, NTRK‐specific inhibitor larotrectinib has been approved by FDA for solid tumor patients with NTRK fusion. Similar with what Sakre N et al reported, we detected RICTOR amplification in 5.7% (7/122) of SCLC patients who might respond to mTORC1/2 inhibitors.^20^ Besides, we found FGFR1 amplifications in 4.9% (6/122) of SCLC patients, and FGFR inhibitors are currently being tested in such patients.^21^ Although the mutation events of these targetable genes are not very common, due to the fact that lung cancers are very prevalent and numerous, even small subgroups represent a large number of patients in need of therapy in China and worldwide.^22^

We also compared our genomic profiling data with that in other cohort. We know that NOTCH‐ASCL1‐RB‐p53 signaling axis driving and maintaining the phenotype of small cell cancers (SCCs) and bi‐allelic inactivation of TP53 and RB1 is characteristic for a SCC phenotype.^11^, ^23^ In Chinese SCLC patients, even though the co‐occurrence of *TP53* and *RB1* alterations was still high (74.6%), it was lower than East Asia patients cohort (82%),^24^ and Western patients cohort (90.9%).^11^ This suggest that in addition to TP53 and RB1, more obligatory tumor suppressor genes such as ARID1A and APC,^25^, ^26^ which showed higher mutation rates in Chinese SCLC, may play essential role in Chinese SCLC. Interestingly, in this study, we also found that the alterations in *EGFR*, *TERT*, *NOTCH1*, *TNFSF13B*, and *BRCA2* genes were only present in the *TP53*/*RB1* co‐alterations patients, and *FGFR1* amplification was more common in patients with *TP53* mutation alone. However, the molecular mechanisms of these remain unclear.

Our genomic analyses further compared the genetic alterations involved in several cancer‐related signaling pathways between Chinese cohort and Western cohort. We identified that the mutation rates of genes related with Wnt, HR, and Notch signaling pathway were lower in Chinese SCLC patients than those in Westerner patients. In contrast, the detection rate of cell cycle‐related genes alterations was higher in Chinese SCLC patients than that in Western patients. Furthermore, we analyzed the basic characteristics of *Notch*,* CREBBP*, and *EP300* gene alterations in Chinese SCLC patients as all three genes have been identified as recurrent mutations in SCLC and may be related to therapy effects.^11^, ^27^ We found that the mutation frequencies of Notch, CREBBP, and EP300 were much lower than those of the reported previously. Although the clinical significance and mechanism of these differences are unclear, these differences should be taken into consideration when developing new treatment strategy targeting Chinese SCLC patients.^28^

Moreover, several studies including ours mentioned that the SCLC tumors had a significantly high mutation rate (TMB‐H), which is considered as a biomarker of response to immunotherapy in SCLC, and immunotherapy did improve the treatment of SCLC.^29^ However, the response rate of patients with SCLC to immunotherapy is much lower than the proportion of patients with TMB‐H, which suggested that TMB alone does not clearly discriminate all responders from nonresponders and more biomarkers may be required in SCLC.^30^ In this study, we found that the mutation rates of several genes such as *FAT1*,* TP53*,* SPTA1*,* KEAP1*,* KMT2D*,* MAGI2*,* NOTCH2*,* NOTCH3*,* FLT1*,* KDM6A*, and *FAT4* in TMB‐H group were significantly higher than those in TMB‐L group. The significance of this phenomenon is much less clear and functional experiments will be required, but it may provide more possibilities on developing new factors that predict response to immune checkpoint inhibitor therapy.

## 5 CONCLUSIONS

In this study, we characterized the genomic alteration profile of Chinese SCLC patients. Consistent with previous report, high mutation rates of *TP53* and *RB1* are the most important genomic features of SCLC, but the detection rate had a certain difference compared with previous study which might be mainly enrolled the Western patients. The detection rate of several gene alterations such as Wnt, and Notch pathway was also different from previous study. These results suggested that the molecular mechanism of SCLC in most Chinese patients was same as that in Western patients, but a few of them had their own unique characteristics. This might attract attention when developing targeted therapy for SCLC patients in China.

## CONFLICT OF INTEREST

Yuan Zhang, Yanfei Yu, Hui Chen, Kuai Liu, Ming Yao, and Kai Wang are employees of OrigiMed.

## AUTHOR CONTRIBUTIONS

Jing Hu, Yu Wang were involved in conceptualization, data curation, formal analysis, investigation, project administration, writing, reviewing, and editing the original draft. Yuan Zhang, Yanfei Yu, Kuai Liu, Ming Yao, Kai Wang were involved in data curation, formal analysis, investigation, methodology, software, and visualization. Weiguang Gu was involved in conceptualization, data curation, project administration, resources, supervision, validation, writing, reviewing, and editing the original draft. Tao Shou involved in conceptualization, funding acquisition, project administration, resources, supervision, validation, writing, reviewing, and editing the original draft.

## References

[1] Govindan R , Page N , Morgensztern D , et al. Changing epidemiology of small‐cell lung cancer in the United States over the last 30 years: analysis of the surveillance, epidemiologic, and end results database. J Clin Oncol. 2006;24(28):4539‐4544. 17008692 10.1200/JCO.2005.04.4859

[2] Kalemkerian GP , Akerley W , Bogner P , et al. Small cell lung cancer. J Natl Compr Canc Netw. 2013;11(1):78‐98. 23307984 10.6004/jnccn.2013.0011 PMC3715060

[3] Califano R , Abidin AZ , Peck R , Faivre‐Finn C , Lorigan P . Management of small cell lung cancer: recent developments for optimal care. Drugs. 2012;72(4):471‐490. 22356287 10.2165/11597640-000000000-00000

[4] Mok TS , Wu Y‐L , Thongprasert S , et al. Gefitinib or carboplatin‐paclitaxel in pulmonary adenocarcinoma. N Engl J Med. 2009;361(10):947‐957. 19692680 10.1056/NEJMoa0810699

[5] Rosell R , Carcereny E , Gervais R , et al. Erlotinib versus standard chemotherapy as first‐line treatment for European patients with advanced EGFR mutation‐positive non‐small‐cell lung cancer (EURTAC): a multicentre, open‐label, randomised phase 3 trial. Lancet Oncol. 2012;13(3):239‐246. 22285168 10.1016/S1470-2045(11)70393-X

[6] Shaw AT , Kim DW , Nakagawa K , et al. Crizotinib versus chemotherapy in advanced ALK‐positive lung cancer. N Engl J Med. 2013;368(25):2385‐2394. 23724913 10.1056/NEJMoa1214886

[7] Kannaiyan R , Mahadevan D . A comprehensive review of protein kinase inhibitors for cancer therapy. Expert Rev Anticancer Ther. 2018;18(12):1249‐1270. 30259761 10.1080/14737140.2018.1527688 PMC6322661

[8] Kandoth C , McLellan MD , Vandin F , et al. Mutational landscape and significance across 12 major cancer types. Nature. 2013;502(7471):333‐339. 24132290 10.1038/nature12634 PMC3927368

[9] Ross JS , Wang K , Elkadi OR , et al. Next‐generation sequencing reveals frequent consistent genomic alterations in small cell undifferentiated lung cancer. J Clin Pathol. 2014;67(9):772‐776. 24978188 10.1136/jclinpath-2014-202447 PMC4145440

[10] Takahashi T , Obata Y , Sekido Y , et al. Expression and amplification of myc gene family in small cell lung cancer and its relation to biological characteristics. Cancer Res. 1989;49(10):2683‐2688. 2540905

[11] George J , Lim JS , Jang SJ , et al. Comprehensive genomic profiles of small cell lung cancer. Nature. 2015;524(7563):47‐53. 26168399 10.1038/nature14664 PMC4861069

[12] Meyerson M , Gabriel S , Getz G . Advances in understanding cancer genomes through second‐generation sequencing. Nat Rev Genet. 2010;11(10):685‐696. 20847746 10.1038/nrg2841

[13] Karachaliou N , Sosa AE , Rosell R . Unraveling the genomic complexity of small cell lung cancer. Transl Lung Cancer Res. 2016;5(4):363‐366. 27650513 10.21037/tlcr.2016.07.02 PMC5009089

[14] Cowin PA , George J , Fereday S , et al. LRP1B deletion in high‐grade serous ovarian cancers is associated with acquired chemotherapy resistance to liposomal doxorubicin. Cancer Res. 2012;72(16):4060‐4073. 22896685 10.1158/0008-5472.CAN-12-0203

[15] Tabouret E , Labussiere M , Alentorn A , Schmitt Y , Marie Y , Sanson M . LRP1B deletion is associated with poor outcome for glioblastoma patients. J Neurol Sci. 2015;358(1–2):440‐443. 26428308 10.1016/j.jns.2015.09.345

[16] Lv S , Ji L , Chen B , et al. Histone methyltransferase KMT2D sustains prostate carcinogenesis and metastasis via epigenetically activating LIFR and KLF4. Oncogene. 2018;37(10):1354‐1368. 29269867 10.1038/s41388-017-0026-x PMC6168472

[17] Helsten T , Kato S , Schwaederle M , et al. Cell‐cycle gene alterations in 4,864 tumors analyzed by next‐generation sequencing: implications for targeted therapeutics. Mol Cancer Ther. 2016;15(7):1682‐1690. 27196769 10.1158/1535-7163.MCT-16-0071

[18] Cui M , Augert A , Rongione M , et al. PTEN is a potent suppressor of small cell lung cancer. Mol Cancer Res. 2014;12(5):654‐659. 24482365 10.1158/1541-7786.MCR-13-0554 PMC4020961

[19] Zhu YC , Liao XH , Wang WX , et al. Patients harboring ALK rearrangement adenocarcinoma after acquired resistance to crizotinib and transformation to small‐cell lung cancer: a case report. Onco Targets Ther. 2017;10:3187‐3192. 28721068 10.2147/OTT.S139718 PMC5499929

[20] Sakre N , Wildey G , Behtaj M , et al. RICTOR amplification identifies a subgroup in small cell lung cancer and predicts response to drugs targeting mTOR. Oncotarget. 2017;8(4):5992‐6002. 27863413 10.18632/oncotarget.13362 PMC5351607

[21] Weiss J , Sos ML , Seidel D , et al. Frequent and focal FGFR1 amplification associates with therapeutically tractable FGFR1 dependency in squamous cell lung cancer. Sci Transl Med. 2010;2(62):62ra93. 10.1126/scitranslmed.3001451 PMC3990281 21160078

[22] Rolfo C , Raez L . New targets bring hope in squamous cell lung cancer: neurotrophic tyrosine kinase gene fusions. Lab Invest. 2017;97(11):1268‐1270. 29085074 10.1038/labinvest.2017.91

[23] Meder L , König K , Ozretić L , et al. NOTCH, ASCL1, p53 and RB alterations define an alternative pathway driving neuroendocrine and small cell lung carcinomas. Int J Cancer. 2016;138(4):927‐938. 26340530 10.1002/ijc.29835 PMC4832386

[24] Lee J‐K , Lee J , Kim S , et al. Clonal history and genetic predictors of transformation into small‐cell carcinomas from lung adenocarcinomas. J Clin Oncol. 2017;35(26):3065‐3074. 28498782 10.1200/JCO.2016.71.9096

[25] Zhang YI , Xu X , Zhang M , et al. ARID1A is downregulated in non‐small cell lung cancer and regulates cell proliferation and apoptosis. Tumour Biol. 2014;35(6):5701‐5707. 24566899 10.1007/s13277-014-1755-x

[26] Ohgaki H , Kros JM , Okamoto Y , Gaspert A , Huang H , Kurrer MO . APC mutations are infrequent but present in human lung cancer. Cancer Lett. 2004;207(2):197‐203. 15072829 10.1016/j.canlet.2003.10.020

[27] Peifer M , Fernández‐Cuesta L , Sos ML , et al. Integrative genome analyses identify key somatic driver mutations of small‐cell lung cancer. Nat Genet. 2012;44(10):1104‐1110. 22941188 10.1038/ng.2396 PMC4915822

[28] Umemura S , Tsuchihara K , Goto K . Genomic profiling of small‐cell lung cancer: the era of targeted therapies. Jpn J Clin Oncol. 2015;45(6):513‐519. 25670763 10.1093/jjco/hyv017

[29] Boumber Y . Tumor mutational burden (TMB) as a biomarker of response to immunotherapy in small cell lung cancer. J Thorac Dis. 2018;10(8):4689‐4693. 30233840 10.21037/jtd.2018.07.120 PMC6129910

[30] Havel JJ , Chowell D , Chan TA . The evolving landscape of biomarkers for checkpoint inhibitor immunotherapy. Nat Rev Cancer. 2019;19(3):133‐150. 30755690 10.1038/s41568-019-0116-x PMC6705396

