# Supplementary material for: Comprehensive genomic profiling of small cell lung cancer in Chinese patients and the implications for therapeutic potential
Source: Cancer Med. 2019 Jun 14;8(9):4338–47. doi: 10.1002/cam4.2199 (PMC6675718; doi:10.1002/cam4.2199)
Supplement: Supplementary file 1 [file CAM4-8-4338-s001.docx]

**Supplementary table 1: All the genes mutated in patients with ETV6-NTRK3 fusion**

| **Patient** | **Mutated genes** | | | **Variation** | **Frequency** | | | **TMB** |
| --- | --- | --- | --- | --- | --- | --- | --- | --- |
| Patient 1 | *ETV6-NTRK3*  *TP53*  *RB1*  *EGFR*  *CDH1*  *KMT2D*  *NFE2L2*  *SPTA1*  *TOP1*  *EPHB1*  *PARP1*  *BCOR*  *ALK*  *STK24*  *CCNE1*  *IKZF1*  *EGFR* | Fusion  Y220H  F755V  E746_A750del  N56S  A2094I  T260Pfs*8  R1811*  I377V  S481Wfs*44  P369A  S992C  S1020P  Amp  Amp  Amp  Amp | | | -  0.937  0.886  0.839  0.452  0.432  0.291  0.290  0.270  0.131  0.121  0.058  0.020  -  -  -  - | | | 17.6 Muts/Mb |
| Patient 2 | *ETV6-NTRK3*  *NOTCH1*  *RANBP2*  *LRP1B*  *BRCA2*  *BRCA1*  *MAP2K1*  *ATM*  *AXIN1*  *AXIN1*  *TP53*  *KDM5A*  *TSC1*  *FANCM*  *PRKCI*  *RUNX1*  *TIE1*  *FOXP1*  *NRG3*  *DICER1*  *TP53*  *EP300*  *ATRX*  *LRP1B*  *SLIT2*  *RB1*  *MET*  *PRKDC*  *ARAF*  *CDC73*  *FLT4*  *KMT2C*  *KMT2C*  *APC*  *NOTCH1* | | Fusion  G1157R  R198H  c.1971-11T>AS2697N  Y856H  Q354H  Q1361R  P754L  E406*  P151S  M384I  L72V  P1340T  c.450+3A>G  R232W  G724W  H326Y  A414S  R490S  D281Y  D1399Y  E303*  P3301S  R462L  c.1814+2T>C  N1138I  G3702C  H421R  c.423+1G>T  R318L  G315S  R380L  R216Q  P877L | | | -  0.492  0.449  0.441  0.436  0.386  0.383  0.374  0.285  0.179  0.138  0.136  0.13  0.117  0.115  0.113  0.112  0.11  0.102  0.1  0.097  0.094  0.092  0.088  0.087  0.069  0.064  0.061  0.059  0.052  0.048  0.041  0.039  0.022  0.01 | 48.8 Muts/Mb | |

**Supplementary table2: the pathways and genes involved in YuanSuTM450 panel**

| **Pathway** | **Genes involved** |
| --- | --- |
| Cell cycle | *CCND1, CCND2, CCND3, CCNE1, CDC73, CDK12, CDK4, CDK6, CDK8, CDKN1A, CDKN1B, CDKN2A, CDKN2B, CHEK1, CHEK2, MDM2, MDM4, PRKDC, RB1, WEE1, WEE2* |
| Wnt | *APC, AXIN1, AXIN2, CSNK1A1, CTNNA1, CTNNB1, GSK3B, LRP1, LRP1B, LRP2, SMAD2, SMAD3, SMAD4* |
| PI3K-AKT-mTOR | *AKT1, AKT2, FBXW7, PDK1, PIK3C2B, PIK3CA, PIK3CB, PIK3CD, PIK3CG, PIK3R1, PIK3R2, PREX2, PRKACA, PRKAR1A, PTEN, RICTOR, RPTOR, STK11, TSC1, TSC2* |
|  |  |
| HR | *ATM, BAP1, BARD1, BLM, BRCA1, BRCA2, BRIP1, FANCA, FANCC, FANCD2, FANCE, FANCF, FANCG, FANCL, FANCM, PALB2, RAD50, RAD51, RAD51B, RAD51C, RAD51D, RAD52, RAD54B, RAD54L, RECQL, XRCC2, XRCC3* |
| Notch | *EP300, NOTCH1, NOTCH2, NOTCH3, NOTCH4* |
| MAPK | *ARAF, BRAF, CREB3L1, CREB3L2, CREBBP, HRAS, KRAS, MAP2K1, MAP2K2, MAP2K4, MAP3K1, MAP3K13, MAP4K5, MYC, NF1, NRAS, RAF1* |
| ERBB | *EGF, EGFR, ERBB2, ERBB3, ERBB4, IGF1R, IGF2* |
| Estrogen | *ESR1, FOS, GNAQ, NCOA2, NCOR1, SRC* |

**Supplementary Table 3: Association between TMB-H and gene mutations in the Chinese SCLC populations**

| **Gene** | **Mut in TMB-H (N)** | **Wild type in TMB-H (N)** | **Mut in TMB-L (N)** | **Wild type in TMB-L (N)** | **P Value** |
| --- | --- | --- | --- | --- | --- |
| FAT1 | 8 | 48 | 1 | 65 | 0.0083 |
| TP53 | 55 | 1 | 57 | 9 | 0.0166 |
| SPTA1 | 10 | 46 | 3 | 63 | 0.0181 |
| KEAP1 | 5 | 51 | 0 | 66 | 0.0184 |
| KMT2D | 13 | 43 | 6 | 60 | 0.029 |
| MAGI2 | 4 | 52 | 0 | 66 | 0.0418 |
| NOTCH2 | 4 | 52 | 0 | 66 | 0.0418 |
| NOTCH3 | 4 | 52 | 0 | 66 | 0.0418 |
| FLT1 | 4 | 52 | 0 | 66 | 0.0418 |
| KDM6A | 4 | 52 | 0 | 66 | 0.0418 |
| FAT4 | 7 | 49 | 2 | 64 | 0.0491 |

**Supplementary Table 4: Correlation between gene mutation and clinical stages of Chinese SCLC patients**

| **GENE** | | **Stage Ⅲ+Ⅳ** | | | | **StageⅠ+Ⅱ** | | | | | | **P Value** | | **P Value**  **(BH correction)** |
| --- | --- | --- | --- | --- | --- | --- | --- | --- | --- | --- | --- | --- | --- | --- |
|  |  | **mut (N)** | **wild type(N)** | | **Freq** | **mut (N)** | | **wild type(N)** | | **Freq** | |  |  |  |
| EPHA3 | | 1 | 38 | | 2.6% | 3 | | 9 | | 25.0% | | 0.0363 | | 1 |
| NOTCH2 | | 1 | 38 | | 2.6% | 3 | | 9 | | 25.0% | | 0.0363 | | 1 |
| TP53 | | 38 | 1 | | 97.4% | 9 | | 3 | | 75.0% | | 0.0363 | | 1 |
| BTK | | 0 | 39 | | 0.0% | 2 | | 10 | | 16.7% | | 0.0518 | | 1 |
| DDR2 | | 0 | 39 | | 0.0% | 2 | | 10 | | 16.7% | | 0.0518 | | 1 |
| INHBA | | 0 | 39 | | 0.0% | 2 | | 10 | | 16.7% | | 0.0518 | | 1 |
| FGF10 | | 3 | 36 | | 7.7% | 3 | | 9 | | 25.0% | | 0.1338 | | 1 |
| SOX2 | | 3 | 36 | | 7.7% | 3 | | 9 | | 25.0% | | 0.1338 | | 1 |
| HCK | | 1 | 38 | | 2.6% | 2 | | 10 | | 16.7% | | 0.1342 | | 1 |
| NRG3 | | 1 | 38 | | 2.6% | 2 | | 10 | | 16.7% | | 0.1342 | | 1 |
| TNFSF13B | | 1 | 38 | | 2.6% | 2 | | 10 | | 16.7% | | 0.1342 | | 1 |
| CIC | | 2 | 37 | | 5.1% | 2 | | 10 | | 16.7% | | 0.2320 | | 1 |
| ETV5 | | 2 | 37 | | 5.1% | 2 | | 10 | | 16.7% | | 0.2320 | | 1 |
| FGF14 | | 2 | 37 | | 5.1% | 2 | | 10 | | 16.7% | | 0.2320 | | 1 |
| IGF1R | | 2 | 37 | | 5.1% | 2 | | 10 | | 16.7% | | 0.2320 | | 1 |
| KDM5A | | 2 | 37 | | 5.1% | 2 | | 10 | | 16.7% | | 0.2320 | | 1 |
| NFE2L2 | | 2 | 37 | | 5.1% | 2 | | 10 | | 16.7% | | 0.2320 | | 1 |
| PIK3CA | | 2 | 37 | | 5.1% | 2 | | 10 | | 16.7% | | 0.2320 | | 1 |
| PRKCI | | 2 | 37 | | 5.1% | 2 | | 10 | | 16.7% | | 0.2320 | | 1 |
| AKT1 | | 0 | 39 | | 0.0% | 1 | | 11 | | 8.3% | | 0.2353 | | 1 |
| ARFRP1 | | 0 | 39 | | 0.0% | 1 | | 11 | | 8.3% | | 0.2353 | | 1 |
| BARD1 | | 0 | 39 | | 0.0% | 1 | | 11 | | 8.3% | | 0.2353 | | 1 |
| BRAF | | 0 | 39 | | 0.0% | 1 | | 11 | | 8.3% | | 0.2353 | | 1 |
| COL1A1 | | 0 | 39 | | 0.0% | 1 | | 11 | | 8.3% | | 0.2353 | | 1 |
| CYP17A1 | | 0 | 39 | | 0.0% | 1 | | 11 | | 8.3% | | 0.2353 | | 1 |
| DDR1 | | 0 | 39 | | 0.0% | 1 | | 11 | | 8.3% | | 0.2353 | | 1 |
| DOT1L | | 0 | 39 | | 0.0% | 1 | | 11 | | 8.3% | | 0.2353 | | 1 |
| ERBB3 | | 0 | 39 | | 0.0% | 1 | | 11 | | 8.3% | | 0.2353 | | 1 |
| FANCE | | 0 | 39 | | 0.0% | 1 | | 11 | | 8.3% | | 0.2353 | | 1 |
| FANCL | | 0 | 39 | | 0.0% | 1 | | 11 | | 8.3% | | 0.2353 | | 1 |
| FANCM | | 0 | 39 | | 0.0% | 1 | | 11 | | 8.3% | | 0.2353 | | 1 |
| FLT1 | | 0 | 39 | | 0.0% | 1 | | 11 | | 8.3% | | 0.2353 | | 1 |
| FOXO1 | | 0 | 39 | | 0.0% | 1 | | 11 | | 8.3% | | 0.2353 | | 1 |
| HSD3B1 | | 0 | 39 | | 0.0% | 1 | | 11 | | 8.3% | | 0.2353 | | 1 |
| IRF2 | | 0 | 39 | | 0.0% | 1 | | 11 | | 8.3% | | 0.2353 | | 1 |
| KMT2A | | 0 | 39 | | 0.0% | 1 | | 11 | | 8.3% | | 0.2353 | | 1 |
| PDCD1 | | 0 | 39 | | 0.0% | 1 | | 11 | | 8.3% | | 0.2353 | | 1 |
| PDCD1LG2 | | 0 | 39 | | 0.0% | 1 | | 11 | | 8.3% | | 0.2353 | | 1 |
| PKD2 | | 0 | 39 | | 0.0% | 1 | | 11 | | 8.3% | | 0.2353 | | 1 |
| PMS2 | | 0 | 39 | | 0.0% | 1 | | 11 | | 8.3% | | 0.2353 | | 1 |
| PTK2 | | 0 | 39 | | 0.0% | 1 | | 11 | | 8.3% | | 0.2353 | | 1 |
| PTK6 | | 0 | 39 | | 0.0% | 1 | | 11 | | 8.3% | | 0.2353 | | 1 |
| RAD51 | | 0 | 39 | | 0.0% | 1 | | 11 | | 8.3% | | 0.2353 | | 1 |
| RAD51C | | 0 | 39 | | 0.0% | 1 | | 11 | | 8.3% | | 0.2353 | | 1 |
| RAF1 | | 0 | 39 | | 0.0% | 1 | | 11 | | 8.3% | | 0.2353 | | 1 |
| RXRA | | 0 | 39 | | 0.0% | 1 | | 11 | | 8.3% | | 0.2353 | | 1 |
| SETBP1 | | 0 | 39 | | 0.0% | 1 | | 11 | | 8.3% | | 0.2353 | | 1 |
| SRMS | | 0 | 39 | | 0.0% | 1 | | 11 | | 8.3% | | 0.2353 | | 1 |
| TBX3 | | 0 | 39 | | 0.0% | 1 | | 11 | | 8.3% | | 0.2353 | | 1 |
| TCF7L2 | | 0 | 39 | | 0.0% | 1 | | 11 | | 8.3% | | 0.2353 | | 1 |
| TFE3 | | 0 | 39 | | 0.0% | 1 | | 11 | | 8.3% | | 0.2353 | | 1 |
| TIE1 | | 0 | 39 | | 0.0% | 1 | | 11 | | 8.3% | | 0.2353 | | 1 |
| TIPARP | | 0 | 39 | | 0.0% | 1 | | 11 | | 8.3% | | 0.2353 | | 1 |
| USP6 | | 0 | 39 | | 0.0% | 1 | | 11 | | 8.3% | | 0.2353 | | 1 |
| XRCC3 | | 0 | 39 | | 0.0% | 1 | | 11 | | 8.3% | | 0.2353 | | 1 |
| ERBB4 | | 5 | 34 | | 12.8% | 0 | | 12 | | 0.0% | | 0.3231 | | 1 |
| KDM6A | | 5 | 34 | | 12.8% | 0 | | 12 | | 0.0% | | 0.3231 | | 1 |
| TERT | | 5 | 34 | | 12.8% | 3 | | 9 | | 25.0% | | 0.3720 | | 1 |
| AKT3 | | 1 | 38 | | 2.6% | 1 | | 11 | | 8.3% | | 0.4188 | | 1 |
| AMER1 | | 1 | 38 | | 2.6% | 1 | | 11 | | 8.3% | | 0.4188 | | 1 |
| AR | | 1 | 38 | | 2.6% | 1 | | 11 | | 8.3% | | 0.4188 | | 1 |
| ARAF | | 1 | 38 | | 2.6% | 1 | | 11 | | 8.3% | | 0.4188 | | 1 |
| ARID1B | | 1 | 38 | | 2.6% | 1 | | 11 | | 8.3% | | 0.4188 | | 1 |
| ATR | | 1 | 38 | | 2.6% | 1 | | 11 | | 8.3% | | 0.4188 | | 1 |
| BCL2L1 | | 1 | 38 | | 2.6% | 1 | | 11 | | 8.3% | | 0.4188 | | 1 |
| BLM | | 1 | 38 | | 2.6% | 1 | | 11 | | 8.3% | | 0.4188 | | 1 |
| BMX | | 1 | 38 | | 2.6% | 1 | | 11 | | 8.3% | | 0.4188 | | 1 |
| CDH1 | | 1 | 38 | | 2.6% | 1 | | 11 | | 8.3% | | 0.4188 | | 1 |
| CHEK2 | | 1 | 38 | | 2.6% | 1 | | 11 | | 8.3% | | 0.4188 | | 1 |
| FANCA | | 1 | 38 | | 2.6% | 1 | | 11 | | 8.3% | | 0.4188 | | 1 |
| FBXW7 | | 1 | 38 | | 2.6% | 1 | | 11 | | 8.3% | | 0.4188 | | 1 |
| FGFR3 | | 1 | 38 | | 2.6% | 1 | | 11 | | 8.3% | | 0.4188 | | 1 |
| FOS | | 1 | 38 | | 2.6% | 1 | | 11 | | 8.3% | | 0.4188 | | 1 |
| GLI1 | | 1 | 38 | | 2.6% | 1 | | 11 | | 8.3% | | 0.4188 | | 1 |
| GNAS | | 1 | 38 | | 2.6% | 1 | | 11 | | 8.3% | | 0.4188 | | 1 |
| INPP4B | | 1 | 38 | | 2.6% | 1 | | 11 | | 8.3% | | 0.4188 | | 1 |
| JAK1 | | 1 | 38 | | 2.6% | 1 | | 11 | | 8.3% | | 0.4188 | | 1 |
| KLHL6 | | 1 | 38 | | 2.6% | 1 | | 11 | | 8.3% | | 0.4188 | | 1 |
| MYB | | 1 | 38 | | 2.6% | 1 | | 11 | | 8.3% | | 0.4188 | | 1 |
| NOTCH4 | | 1 | 38 | | 2.6% | 1 | | 11 | | 8.3% | | 0.4188 | | 1 |
| NRG1 | | 1 | 38 | | 2.6% | 1 | | 11 | | 8.3% | | 0.4188 | | 1 |
| PARP1 | | 1 | 38 | | 2.6% | 1 | | 11 | | 8.3% | | 0.4188 | | 1 |
| PIK3CB | | 1 | 38 | | 2.6% | 1 | | 11 | | 8.3% | | 0.4188 | | 1 |
| PIK3R1 | | 1 | 38 | | 2.6% | 1 | | 11 | | 8.3% | | 0.4188 | | 1 |
| RET | | 1 | 38 | | 2.6% | 1 | | 11 | | 8.3% | | 0.4188 | | 1 |
| SOX9 | | 1 | 38 | | 2.6% | 1 | | 11 | | 8.3% | | 0.4188 | | 1 |
| SS18 | | 1 | 38 | | 2.6% | 1 | | 11 | | 8.3% | | 0.4188 | | 1 |
| TNFAIP3 | | 1 | 38 | | 2.6% | 1 | | 11 | | 8.3% | | 0.4188 | | 1 |
| TPMT | | 1 | 38 | | 2.6% | 1 | | 11 | | 8.3% | | 0.4188 | | 1 |
| TSHR | | 1 | 38 | | 2.6% | 1 | | 11 | | 8.3% | | 0.4188 | | 1 |
| WEE1 | | 1 | 38 | | 2.6% | 1 | | 11 | | 8.3% | | 0.4188 | | 1 |
| FAM135B | | 6 | 33 | | 15.4% | 3 | | 9 | | 25.0% | | 0.4237 | | 1 |
| LRP1B | | 13 | 26 | | 33.3% | 2 | | 10 | | 16.7% | | 0.4700 | | 1 |
| ADAM29 | | 2 | 37 | | 5.1% | 1 | | 11 | | 8.3% | | 0.5612 | | 1 |
| AKT2 | | 2 | 37 | | 5.1% | 1 | | 11 | | 8.3% | | 0.5612 | | 1 |
| ATM | | 2 | 37 | | 5.1% | 1 | | 11 | | 8.3% | | 0.5612 | | 1 |
| ATRX | | 2 | 37 | | 5.1% | 1 | | 11 | | 8.3% | | 0.5612 | | 1 |
| CCND1 | | 2 | 37 | | 5.1% | 1 | | 11 | | 8.3% | | 0.5612 | | 1 |
| EP300 | | 2 | 37 | | 5.1% | 1 | | 11 | | 8.3% | | 0.5612 | | 1 |
| FEV | | 2 | 37 | | 5.1% | 1 | | 11 | | 8.3% | | 0.5612 | | 1 |
| FGF12 | | 2 | 37 | | 5.1% | 1 | | 11 | | 8.3% | | 0.5612 | | 1 |
| FGF3 | | 2 | 37 | | 5.1% | 1 | | 11 | | 8.3% | | 0.5612 | | 1 |
| GATA1 | | 2 | 37 | | 5.1% | 1 | | 11 | | 8.3% | | 0.5612 | | 1 |
| GLI3 | | 2 | 37 | | 5.1% | 1 | | 11 | | 8.3% | | 0.5612 | | 1 |
| GRM3 | | 2 | 37 | | 5.1% | 1 | | 11 | | 8.3% | | 0.5612 | | 1 |
| IKBKE | | 2 | 37 | | 5.1% | 1 | | 11 | | 8.3% | | 0.5612 | | 1 |
| IKZF1 | | 2 | 37 | | 5.1% | 1 | | 11 | | 8.3% | | 0.5612 | | 1 |
| KEAP1 | | 2 | 37 | | 5.1% | 1 | | 11 | | 8.3% | | 0.5612 | | 1 |
| KIT | | 4 | 35 | | 10.3% | 0 | | 12 | | 0.0% | | 0.5612 | | 1 |
| MAP3K13 | | 2 | 37 | | 5.1% | 1 | | 11 | | 8.3% | | 0.5612 | | 1 |
| MED12 | | 2 | 37 | | 5.1% | 1 | | 11 | | 8.3% | | 0.5612 | | 1 |
| NKX2-1 | | 4 | 35 | | 10.3% | 0 | | 12 | | 0.0% | | 0.5612 | | 1 |
| NOTCH3 | | 2 | 37 | | 5.1% | 1 | | 11 | | 8.3% | | 0.5612 | | 1 |
| NTRK1 | | 4 | 35 | | 10.3% | 0 | | 12 | | 0.0% | | 0.5612 | | 1 |
| NTRK3 | | 4 | 35 | | 10.3% | 0 | | 12 | | 0.0% | | 0.5612 | | 1 |
| PAK3 | | 2 | 37 | | 5.1% | 1 | | 11 | | 8.3% | | 0.5612 | | 1 |
| PDGFRB | | 2 | 37 | | 5.1% | 1 | | 11 | | 8.3% | | 0.5612 | | 1 |
| PIK3C2B | | 2 | 37 | | 5.1% | 1 | | 11 | | 8.3% | | 0.5612 | | 1 |
| PTEN | | 2 | 37 | | 5.1% | 1 | | 11 | | 8.3% | | 0.5612 | | 1 |
| RUNX1T1 | | 2 | 37 | | 5.1% | 1 | | 11 | | 8.3% | | 0.5612 | | 1 |
| SLIT2 | | 2 | 37 | | 5.1% | 1 | | 11 | | 8.3% | | 0.5612 | | 1 |
| SMAD2 | | 2 | 37 | | 5.1% | 1 | | 11 | | 8.3% | | 0.5612 | | 1 |
| TAF1 | | 2 | 37 | | 5.1% | 1 | | 11 | | 8.3% | | 0.5612 | | 1 |
| EPHA5 | | 3 | 36 | | 7.7% | 2 | | 10 | | 16.7% | | 0.5798 | | 1 |
| GRIN2A | | 3 | 36 | | 7.7% | 2 | | 10 | | 16.7% | | 0.5798 | | 1 |
| ROCK1 | | 3 | 36 | | 7.7% | 2 | | 10 | | 16.7% | | 0.5798 | | 1 |
| SDHA | | 3 | 36 | | 7.7% | 2 | | 10 | | 16.7% | | 0.5798 | | 1 |
| IL7R | | 4 | 35 | | 10.3% | 2 | | 10 | | 16.7% | | 0.6164 | | 1 |
| IRS2 | | 4 | 35 | | 10.3% | 2 | | 10 | | 16.7% | | 0.6164 | | 1 |
| LRP1 | | 4 | 35 | | 10.3% | 2 | | 10 | | 16.7% | | 0.6164 | | 1 |
| NOTCH1 | | 4 | 35 | | 10.3% | 2 | | 10 | | 16.7% | | 0.6164 | | 1 |
| ROS1 | | 4 | 35 | | 10.3% | 2 | | 10 | | 16.7% | | 0.6164 | | 1 |
| APC | | 5 | 34 | | 12.8% | 2 | | 10 | | 16.7% | | 0.6618 | | 1 |
| FAT1 | | 5 | 34 | | 12.8% | 2 | | 10 | | 16.7% | | 0.6618 | | 1 |
| MAGI2 | | 5 | 34 | | 12.8% | 2 | | 10 | | 16.7% | | 0.6618 | | 1 |
| STK24 | | 5 | 34 | | 12.8% | 2 | | 10 | | 16.7% | | 0.6618 | | 1 |
